# Knowledge, confidence and social support: Kenyan women’s priority needs for contraceptive self-injection learning through a social cognitive theory lens

**DOI:** 10.1186/s12905-025-03801-4

**Published:** 2025-06-30

**Authors:** Serah Gitome, Lauren Suchman, Sarah Okumu, Pauline Wekesa, Janelli Vallin, Louisa Ndunyu, Zachary A. Kwena, Jenny Liu, Kelsey Holt, Elizabeth A. Bukusi

**Affiliations:** 1 Centre for Microbiology Research, Kenya Medical Research Institute, Nairobi, Kenya; 2 Department of Social and Behavioral Sciences, Institute for Health & Aging, School of Nursing, University of California, San Francisco, San Francisco, CA USA; 3 School of Public Health & Community Development, Maseno University, Kisumu, Kenya; 4 Department of Family and Community Medicine, School of Medicine, University of California, San Francisco, San Francisco, CA USA; 5 Departments of Global Health and Obstetrics and Gynecology, University of Washington, Seattle, WA USA; 6 Department of Obstetrics, Gynecology and Reproductive. Sciences, University of California San Francisco, San Francisco, USA

**Keywords:** Contraceptive self-injection, Self-care, Learning needs, DMPA-SC, Social cognitive theory, Kenya

## Abstract

**Background:**

Self-injectable contraceptives provide individuals the opportunity to prevent pregnancy with a self-controlled method that helps enhance self-care. Kenya is in the process of making self-injectable subcutaneous depot medroxyprogesterone acetate (DMPA-SC) widely available. We know little about what would make women in Kenya feel that contraceptive self-injection (SI) is feasible. Applying the social cognitive theory, we sought to understand what SI-naïve reproductive-aged women wanted to learn about SI, how they desired to learn it, and with whom.

**Methods:**

We conducted sixty-one in-depth interviews with women aged 15–45 years, residing in Nairobi and Kisumu, Kenya. Participants were purposively sampled to ensure diverse representation by age, contraceptive use or non-use, and previous experience with SI of DMPA-SC. Audio recordings of the in-depth interviews were transcribed and translated into English as necessary. Transcripts were coded in Dedoose qualitative analysis software, data was analyzed thematically, and insights were drawn from the emerging themes.

**Results:**

Participants’ SI learning perceptions were influenced by their perceived ability to self-inject, shaped by an interplay of their knowledge of SI (cognitive factors), vis-à-vis their confidence to self-inject (behavioral factors), and their perceived value of social reinforcements to enable the behavior (environmental factors). Participants had low knowledge and skills in SI resulting in low self-efficacy; thus, they preferred direct observational learning from a knowledgeable healthcare provider and practical demonstrations to enhance confidence in their SI skills. In addition, participants desired to learn with someone familiar or close to them who would provide support to navigate the complexities of SI beyond the initial training session.

**Conclusion:**

Our study highlights Kenyan women’s desire for a contraceptive SI learning experience that imparts knowledge through comprehensive counseling and instills confidence through observational learning from healthcare providers and ample practice opportunities until the users feel ready to self-inject at home. By aligning with the WHO self-care guidelines and leveraging the social cognitive theory, client training programs can equip women to adopt SI confidently should they so choose.

**Supplementary Information:**

The online version contains supplementary material available at 10.1186/s12905-025-03801-4.

## Introduction

Self-care refers to the ability of an individual or community to promote or maintain health, prevent disease, or cope with illness or disability with or without support from a healthcare worker [1]. SI of subcutaneous depot medroxyprogesterone acetate (DMPA-SC) may enhance access to contraceptive self-care due to its favorable safety and efficacy profile, and the convenience, autonomy, and privacy it offers to the user [2–13]. The World Health Organization recommends providing self-injectable DMPA-SC as a self-care intervention in an environment that offers appropriate information and training to enable women to make an informed choice [1]. However, there is limited evidence on what level of support or education women need to confidently choose this self-care option.

Evidence shows that training of women in contraceptive SI – featuring face-to-face demonstrations by a trained healthcare provider, hands-on practice on a model and self, and provision of up to three cycles of re-injection supplies for those who demonstrate proficiency – is feasible and acceptable [3, 6, 8, 14–16]. In addition, the all-in-one BD Uniject™ device makes it easy to self-inject DMPA-SC [17]. However, studies in sub-Saharan Africa show that some trained women on SI prefer provider-administered injection due to needle fear, self-harm concerns, and low confidence in their injecting skills [10, 18–20]. Across different countries, women's considerations for contraceptive SI training differ by their desired level of detailed information, training location (community- versus health facility-based), and extent of skills support needed to influence their decision to adopt SI [18, 20–22]. To support women’s adoption and continuation of contraceptive SI, it is essential to understand country-specific learning needs and preferences and tailor the training information accordingly [18, 23].

In 2020, Kenya launched a national DMPA-SC implementation and scale-up plan followed by a pilot introduction of DMPA-SC in a few counties to inform SI practices and subsequent training of various healthcare cadres on the provision of DMPA-SC for SI [24, 25]. Following the adoption of national guidelines on reproductive health self-care [26], Kenya is in the process of making DMPA-SC for SI available countrywide at both public and private facilities [27]. These guidelines recognize that key components of an enabling environment for self-care include information, psychosocial support, and a trained health workforce, among others [26]. Although injectables are among the most commonly used contraceptive methods in Kenya, only about 10% of injectable users chose DMPA-SC, and less than 1% self-injected as of 2022 [28]. Little is known about what women perceive as an enabling environment to help them confidently choose and continue with this novel contraceptive technology.

To address this knowledge gap, we conducted in-depth interviews with a diverse group of Kenyan women to understand their preferences and perceptions of information and support needed to help them choose SI of DMPA-SC if it aligned with their preferences. In our analysis, we apply the lens of social cognitive theory, the main construct of which, reciprocal determinism, posits that learning a behavior occurs because of an interplay between cognitive factors, such as knowledge and attitudes, behavioral factors like skills and self-efficacy, and environmental factors like family or peer influence and social norms. These factors determine whether or not women are likely to use contraceptive SI [29]. Recognizing that individuals have the right to choose a contraceptive method that aligns with their unique preferences and that SI of DMPA-SC may not be the right choice for all women, we aimed to elucidate learning needs that help women choose to enact the behavior of SI should they so choose. A better understanding of these learning needs can inform the Kenya Ministry of Health’s rollout of DMPA-SC for SI in Kenya by potentially guiding the design of SI training programs and contributing to the broader goals of universal health coverage and well-being [1].

## Methods

### Study setting

We conducted a qualitative study in Kenya under the Innovations in Choice and Autonomy (ICAN) project to explore how self-injection of DMPA-SC can best meet women’s needs [30]. The ICAN project, led by the University of California, San Francisco and institutions from Kenya, Uganda, Malawi, and Nigeria aims to expand access to a full range of contraceptive options, including DMPA-SC, in the four African countries. In Kenya, researchers from Kenya Medical Research Institute and Maseno University conducted the study in two geographically and culturally diverse areas, namely Kisumu and Nairobi. Kisumu city lies on the shores of Lake Victoria in the western part of Kenya and hosts a thriving fisherfolk community predominantly comprised of people from the Luo ethnic group. Nairobi is the capital city of Kenya and home to people from all ethnic groups, with more than 60% of its population living in informal settlements where access to healthcare, including reproductive services, is limited [31].

### Recruitment and eligibility

Between February and May 2021, we worked with community health promoters to identify potential participants from the community, health facilities, and pharmacies offering contraceptive services in Nairobi and Kisumu. The study team conducted initial eligibility screening and purposively sampled individuals who self-identified as women of reproductive age to ensure diverse representation by age (15–17, 18–25 and 26–45 years), contraceptive use or non-use, marital status, and with or without experience with SI of DMPA-SC. Married participants were defined as those in a heterosexual marriage or cohabiting relationship, while unmarried participants were single due to never marrying, divorce, separation, or widowhood. To understand the needs of all current and potential contraceptive users, we included both groups. Contraceptive users were defined as those currently or previously using any known method to prevent pregnancy, while non-users had never used these methods. Available contraceptive options in the study areas included injectables, pills, implants, intra-uterine devices, condoms, fertility-awareness methods, and tubal ligation. Due to the low prevalence of contraceptive SI in the country at the time of the study [28], most participants did not have any direct exposure to SI of DMPA-SC.

### Data collection

We obtained written informed consent and assent in the participants’ language of preference (English, Dholuo, or Swahili). In-depth interview guides were developed in English, translated into Dholuo and Swahili, then back-translated to verify that original meaning was preserved, and then pilot-tested. Interviews covered topics such as knowledge about SI, experiences using DMPA-SC, and how and what women wanted to learn about DMPA-SC for SI (Appendix 1: Abbreviated in-depth interview guide). Participants were first asked if they had ever seen or used DMPA-SC. Those who had not were shown a sample of the DMPA-SC unit and given a brief description of how it is self-administered before being asked their views (Appendix 2: Description of self-injection of DMPA-SC given to participants). Interviewers explored participants’ preferences for SI learning modes and their reasons for their preferred method of SI learning. Where necessary, interviewers elicited further interest in additional learning modes using examples.

### Data analysis

Experienced qualitative interviewers, fluent in English, Kiswahili and Dholuo and trained on the study, conducted interviews lasting 1–2 h. The interviews were recorded with participants’ written consent. Audio recordings were transcribed verbatim and translated into English where necessary. All interview transcripts were coded using Dedoose Software Version 9 (Sociocultural Research Consultants, LLC, Los Angeles, California). Researchers used a codebook developed both deductively and inductively based on the available literature and themes drawn from a preliminary review of select transcripts. Ten researchers, including social scientists and public health specialists, participated in the coding. Each coder used Dedoose to individually code a transcript, and then the coders came together (virtually) to collaboratively code the same transcript. This was followed by paired and group sessions to standardize code application. Researchers then independently coded assigned transcripts. Details of the analytic process are described elsewhere [30, 32]. For this paper, we generated relevant code reports on women’s SI interest and familiarity, their training preferences, who they wanted to be involved in the SI training, and the questions they wanted answered during the training. We reviewed the code reports for data that illustrated how the interplay between cognitive, behavioral, and environmental factors (key components of the social cognitive theory’s principle of reciprocal determinism) influenced what women wanted to learn about SI. Data analysis was done thematically, and insights were drawn from the emerging themes [33].

### Ethical approval

Ethical approval for the study was obtained from the Kenya Medical Research Institute Scientific and Ethics Review Unit (KEMRI SERU No.4013) and the University of California, San Francisco Institutional Review Board (UCSF IRB #19–29,805). A research permit was obtained from Kenya’s National Commission for Science, Technology, and Innovation (NACOSTI approval # 464,643).

## Results

Of the 61 participants interviewed, 56% resided in Nairobi and 44% in Kisumu (Table 1). Nearly 40% of the respondents were aged between 18–25 years old while 30% were aged 15–17. Sixty-two percent of the participants had a secondary or college level of education. A majority (64%) were married and had children (57%). Most were unemployed (61%) and 34% had never used a modern contraceptive method. Among the sample’s contraceptive users (N = 40), only two (5%) had used DMPA-SC and neither of them had self-injected.

**Table 1:** Socio-demographic characteristics

Characteristic		*N* = 61 (100%)
County of residence	Nairobi	34 (55.7%)
	Kisumu	27 (44.3%)
Age	15–17 years	18 (29.5%)
	18–25 years	24 (39.3%)
	26–45 years	19 (31.1%)
Highest education level attained	No education	4 (6.6%)
	Primary	16 (26.2%)
	Secondary	30 (49.2%)
	College	8 (13.1%)
	Missing	3 (4.9%)
Marital status	Unmarried	22 (36.1%)
	Married	39 (63.9%)
Employment status	Unemployed	37 (60.7%)
	Employed	22 (36.1%)
	Missing	2 (3.3%)
No. of children	No children	26 (42.6%)
	1–3 children	31 (50.8%)
	> 3 children	4 (6.6%)
Ever used contraceptive^a^	Yes DMPA-SC	2 (3.3%)
	Yes (other contraceptives)	38 (62.3%)
	No	21 (34.4%)
County of residence	Nairobi	34 (55.7%)
	Kisumu	27 (44.3%)

^a^Contraceptive – this included any methods that are currently known to prevent pregnancy

**Fig. 1:**
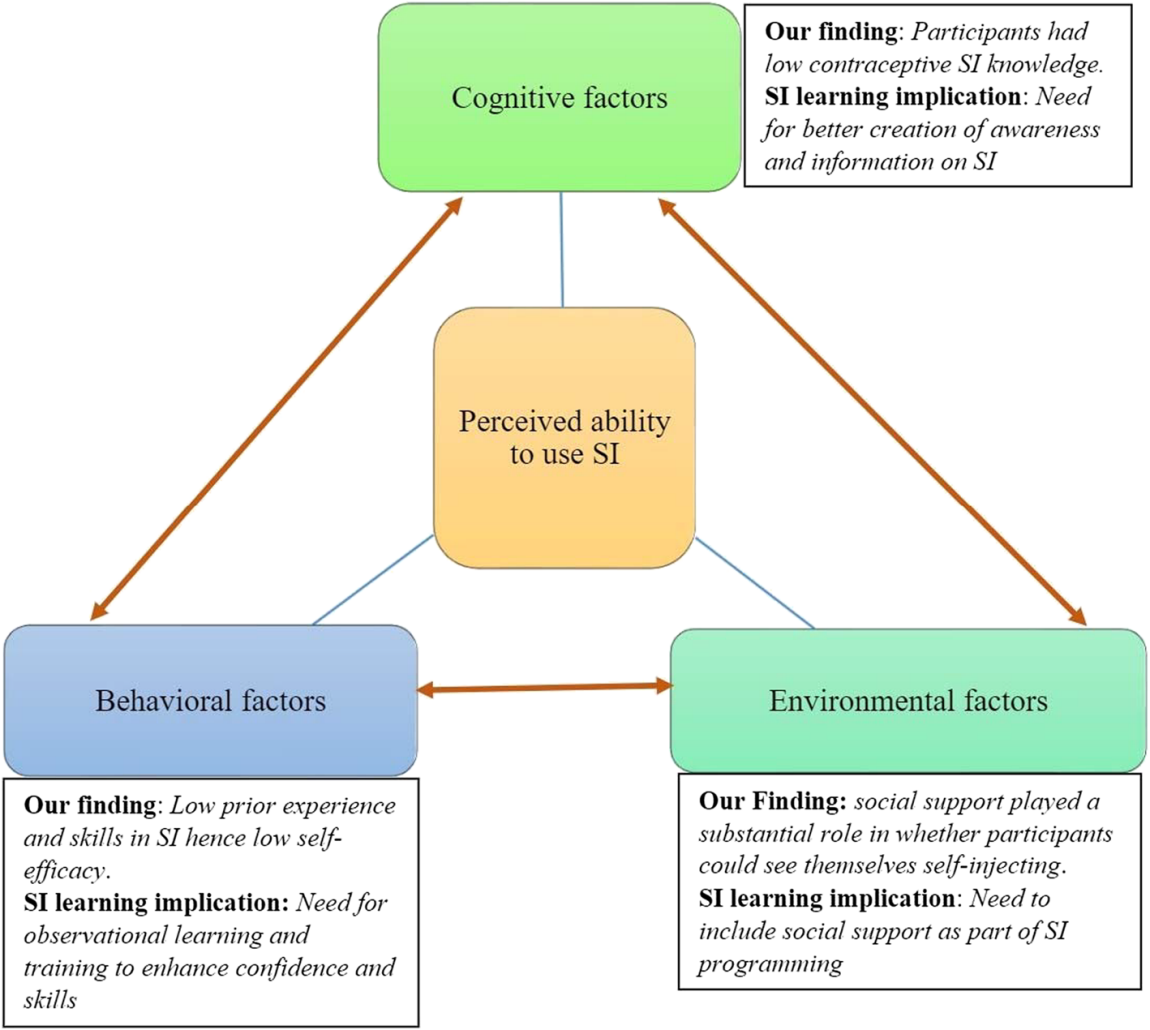
Social Cognitive Theory’s reciprocal determinism applied to DMPA-SC SI learning needs

Aligned with the social cognitive theory’s core concept of reciprocal determinism, participants’ perception of whether they were able to self-inject was shaped by an interplay of their own knowledge of SI (cognitive factors), vis-a-vis their confidence to self-inject (behavioral factors), and their perceived value of social reinforcements to enable the behavior (environmental factors), as illustrated in Fig. 1. This interplay influenced participants’ SI learning needs as discussed under the key themes below.

### Cognitive (knowledge) factors

#### Participants’ understanding of SI varied greatly

Some participants had a general awareness of SI in the context of diabetes management, but most had not seen it in practice and therefore interpreted the idea of SI in different ways, some of which were colored with misunderstanding. Although some correctly understood SI as the act of administering an injection to oneself, to others, SI loosely meant being injected by a lay or non-medical person, usually a close relative. Some participants talked of seeing people self-injecting drugs to manage pain, treat infections like malaria, induce pregnancy termination, or for recreation.

*R:Aah. I do hear just people inject themselves, but I have not even seen [it]. And I am wondering, ‘where do they get that courage to do so?’*

*I:Which one have you ever heard of people injecting themselves with?*

*R:like [for] Malaria. Yes. Or antibiotics.*

(42-year-old contraceptive non-user, Kisumu).

Other participants perceived SI to include self-administered diagnostic blood tests, such as HIV tests, pointing to a low understanding of the distinction between self-administered medications or treatments and tests. Some participants, after receiving an explanation of contraceptive SI, had the misperception that one could overdose on the hormone when self-injecting alone.

*Yes, I would also like to know when now it has overpowered you what can you do because sometimes those drugs(hormonal contraceptives) are very powerful.*

(26-year-old contraceptive non-user, Nairobi)

#### Limited SI knowledge influenced participants'attitudes toward SI and their learning priorities

After being shown a sample DMPA-SC SI unit and a brief description of how it is self-administered, participants’ initial reactions were mixed; some showed interest in self-injecting DMPA-SC and others felt apprehensive about it. Those interested in DMPA-SC appreciated its similarity to the widely used intramuscular DMPA and vocalized the added benefits of SI, such as, convenience of home use, discreetness when administering the method, reduced clinic visits, and ease of use. However, other participants were apprehensive of self-injecting due to a fear of inflicting pain on themselves, a perceived inability to self-inject properly, and a fear that something could go wrong when self-injecting in the absence of a healthcare provider.

*I:If at all you will need a family planning method, would you like to inject yourself with [DMPA-SC]?*

*R:No…Cause I have fear for needles.*

*I:You fear needles? What do you fear about needles?*

*R:The pain… I have a feeling it can break in my body (laughter)*

(16-year-old contraceptive non-user, Nairobi).

Thus, participants discussed questions they would have for healthcare providers about SI mechanics, safety, and effectiveness that would help them feel more confident to self-inject were they to choose this method.

SI mechanics and safety: Participants were curious about the mechanics of SI. They wanted to know more about how to prepare the medicine for injection (e.g., diluting the medicine, putting it in the Uniject™ system, whether to shake it before use, and how to expel the medicine into their bodies). Other information sought included how long it would take for the self-injected contraceptive to start working and how or when to stop DMPA-SC use.

*Like… I would ask if like the drug is. You see? If you inject yourself with that part, do you press there so that the medicine comes out or the medicine is on the tip?*

(17-year-old contraceptive user, Kisumu).

In addition, participants sought to understand which parts of the body to inject themselves and why those parts, what time of day/month to inject themselves, how often to inject themselves, whether to inject each unit once or multiple times, how many units to inject each time they administered the method, and how to dispose of the used unit.

Effectiveness of contraceptive SI: Participants wanted assurance that DMPA-SC was highly effective in preventing pregnancy, comparable to provider-administered DMPA.

*The first thing I would like to know is if it can prevent pregnancy 100%, not that when you use it you are 50-50, I would like to know that if you use it then you are sure that you won’t get pregnant.*

(20-year-old contraceptive user, Kisumu)

They also discussed concerns about the injection site affecting effectiveness with one woman preferring injecting in the buttocks over arms due to the perceived higher efficacy of a gluteal injection compared to one in the arm.

Thus, participants sought information that addressed their concerns about users’ ability to self-inject correctly and safely, and how well DMPA-SC could prevent pregnancy in comparison to provider-administered intramuscular DMPA. These factors weighed on their perceived confidence and their interest in attempting contraceptive SI.

### Behavioral factors

#### Lack of firsthand SI experience

None of the participants had ever self-injected before joining our study. Two participants had received provider-administered DMPA-SC at a pharmacy and were aware that it could be self-administered but had not learned how to self-inject. One of these participants was offered an opportunity to learn SI but she declined out of fear; the other participant was not interested in learning about SI or continuing with the method.

*The one that you can inject yourself… Sayona?... The little I know about it is that I was told that it works just as Depo (DMPA-IM) and with it you can inject yourself that’s the only thing I know about it… I was told that I can take some if I want to though personally I can’t (laughter). I fear injecting myself*.

(32-year-old DMPA-SC user, Nairobi)

#### Participants preferred observational learning from a knowledgeable healthcare provider, emphasizing the need to build confidence in SI

Participants overwhelmingly preferred to learn about contraceptive SI from healthcare providers due to their perceived expertise and ability to provide comprehensive information about the SI process, how it worked, and method side effects. They particularly valued learning from doctors or nurses who could demonstrate the SI process step-by-step until the user mastered the skill.

*If I am with the doctor and he gets me the injection to inject myself and he supervises how I inject myself you know he can teach you… it’s called practicals so that you know like if you put it here he will tell you take it back like this so if I inject myself while he is there and he tells me that I have done it well then I can even go with it if at all I don’t fear injecting myself then I can go with it home and inject myself.*

(32-year-old DMPA-SC user, Nairobi)

Further, participants trusted healthcare facilities as reliable sources of information about contraceptive SI, contrasting them with potentially less reliable sources such as community members.

Participants expressed that learning from healthcare providers would offer a safe environment where new self-injectors could make mistakes and build confidence with professional correction. They also wanted healthcare providers to be available to handle potential injection mishaps, like a needle missing the target site or breaking in the body during SI.

*The question is what if maybe if you are injecting yourself and that needle gets inside, how will you help yourself at that point and you are alone?*

(19-year-old contraceptive non-user, Nairobi)

However, some participants noted that healthcare providers in public facilities were overburdened and might not have adequate time for detailed SI training, leading to insufficient instruction. They preferred learning from well-trained community health volunteers, who could provide thorough SI training and support conveniently at home.

*Because the community workers would walk with the community. They walk with you..... So, if today is so and so’s turn [to self-inject] they book an appointment with you. They come and see, and they support you. So, it is good if they are trained so that they can support us in the community.*

(38-year-old contraceptive user, Kisumu).

Participants generally preferred one-on-one sessions to group sessions so that they would have maximum attention from the healthcare provider and at the same time concentrate on what they were being taught without distractions from fellow learners.

*On lessons for me to understand it will be good when somebody is doing it one-on-one with the person who is going to use it…it will be easier and I will understand it better as compared to when it is done in a group where people don’t concentrate maybe someone wants to whisper something in your ears and you want to concentrate and know how this thing works.*

(30-year-old contraceptive user, Kisumu)

Additionally, participants thought that online videos that provided detailed step-by-step instructions could be useful for reference and generating interest in SI. However, they noted that high internet costs, information inadequacy, and perceived unreliability of online information limited the utility of videos in the learning process.

*Videos; it depends, maybe you’ll have to use the bundles when the bundles finish and maybe you don’t have money you see other information, you’ll not get enough information that you needed.*

(24-year-old contraceptive non-user, Nairobi).

Participants felt that written materials such as pamphlets, posters, and flyers were less useful than learning from a healthcare provider because they had limited capacity to provide comprehensive information, practical demonstrations or interactivity and required a certain level of literacy for one to understand the information.

*You are reading, yes, but you don’t understand. The way people understand things are different among people, it can be [written] “inject here” but you end up injecting it on your thighs (wrong place) so it is better for the medic or clinician to teach you because one, they have studied about it in a class and they did practicals on it.*

(34-year-old contraceptive user, Kisumu)

### Environmental factors

#### Participants desired to learn with familiar individuals who could be a source of continued support to navigate the complexities of SI.

Participants overwhelmingly preferred someone familiar and close to them to be involved in their SI learning journey. The key criteria for co-learners were individuals who knew of or had experience using contraception, could maintain discretion about the participant’s contraceptive use, and could be relied upon to provide continued support for the woman to self-inject before she achieved proficiency. Respondents mentioned preferences for three types of co-learners: male sexual partners, a close family member, and a peer. A minority of respondents expressed preference to learn alone.

Learning with male sexual partners and other close family members: Participants felt male partners would instill confidence to self-inject by providing additional information should the woman forget some of the SI steps, administering the injection until the participant was confident enough to self-inject and supporting decision-making regarding SI use. Younger participants, especially teenagers, preferred learning with their mothers, whom they described as knowledgeable and experienced in family planning and hence could guide them if they made a mistake while injecting themselves.

*So maybe I get someone who is experienced with these things. Yeah, let me just use my mum if maybe she is ever used that, I will go tell her you know mum help me do this and this or you can take me to the doctor so that when I do it the wrong way she will tell me no this is how it is being done.*

* (27-year-old* contraceptive user*, Nairobi).*

Other participants preferred to learn with their children or sisters who could remind them of any SI steps they forgot. Participants also wanted to learn with their sisters, whom they thought could benefit from knowing more about contraceptive SI.

Learning with peers: Close friends were preferred co-learners for participants who wanted to maintain discretion about contraceptive use or those who felt friends, if also trained, could provide additional learning support. Some participants felt that contraception was only a woman’s concern, preferring to learn with female peers, including schoolmates and social media group members, to share knowledge and benefits.

*Because they (friends) will be able to help me because my husband won’t he will keep telling me that I am bothering him with female issues … that’s why I’m saying my friend*

*(17-year-old contraceptive non-user, Nairobi)*

A few participants, mostly young and unmarried, preferred to learn on their own to keep their contraceptive use private. Others felt expert instruction from healthcare providers was sufficient and did not need someone else to learn with.

## Discussion

Our analysis using the social cognitive theory showed that women's SI learning needs were shaped by an interplay of cognitive, behavioral and environmental factors that influenced the likelihood that they would be comfortable choosing SI. First, participants’ limited understanding of SI stemmed from a lack of direct experience with, and some misconceptions about, contraceptive SI (cognitive factors). This low contraceptive SI familiarity contributed to a mix of initial interest and apprehension towards SI (behavioral factors) which prompted participants to desire an observational learning approach that prioritized comprehensiveness of information, healthcare provider guidance, safety, and confidence to self-inject (behavioral factors). In addition, participants desired strong social support from close family members and friends to navigate the complexities of SI (environmental factors).

### Cognitive (Knowledge) factors

Most participants lacked a fundamental understanding of contraceptive SI. Thus, they felt they needed to learn more before they could feel comfortable self-injecting, implying that providing contraceptive SI is not as simple as giving women access to the method [14, 22, 34]. Fear of needles, lack of confidence, and the perception that SI meant managing one’s contraceptive needs alone deepened women’s interest in getting the mechanics of SI right to minimize the risk of self-harm. The initial fear of SI as a barrier to the uptake of contraceptive SI has been described in numerous studies in sub-Saharan Africa and globally and takes many forms, such as fear of needles, fear of inflicting pain or harm, and fear of self-injecting incorrectly [14, 15, 18, 35]. Similar to our findings, research from a human-centered design process in Uganda and Nigeria showed that women seek comprehensive information, including contraceptive counseling and logistical details, to decide if self-injectable contraceptives will help them achieve their family planning goals [21]. Equipping healthcare providers to counsel comprehensively, coupled with targeted training that addresses initial fears, aligns with WHO’s and the Kenya Ministry of Health’s emphasis on the necessity of accessible information to empower women to autonomously choose contraceptive SI, and boost their confidence and proficiency in using the method [14, 23, 26, 36].

Many SI programs today emphasize on providing written instructional aides to the client [18, 36–38], but women in our study felt that written materials would have limited value in their learning journey due to limitations of literacy and lack of interactivity. There is a need to explore other ways to support women to learn, such as the use of well-trained community health workers or volunteers because they tend to be a trusted source of contraceptive information and products in many communities and can access harder-to-reach women [39]. Online videos, though perceived by the participants to be supplementary to the initial provider-led training, may confer enhanced utility if they encompass interactive features that allow real-time questions and responses, thus aligning with the valued attributes of face-to-face learning. Online videos may be attractive to women who want to self-inject discreetly and are afraid that printed materials will be found by others in the household. Making use of innovative user learning methods to combine healthcare provider-led training with other approaches such as online videos aligns with the WHO's endorsement of diverse educational strategies to meet women’s different learning needs and preferences [1]. In line with Bandura’s social cognitive theory, equipping women with the requisite SI knowledge in the manner that best supports their learning preferences can potentially boost their self-efficacy to successfully initiate and continue using this SI method if they so choose [34, 40].

### Behavioral factors

Although SI is primarily a self-care method, the healthcare worker was very much a present figure in the women’s desired learning journey, whether this was explicitly stated (e.g. most participants preferred to learn about SI from healthcare providers) or implied (e.g. participants’ anxiety about something going wrong when injecting in the absence of a healthcare provider). Participants preferred to learn by first observing as the provider demonstrated how to self-inject in a stepwise manner and then practicing under the provider’s supervision and guidance. Observational learning is a key principle outlined in the social cognitive theory which emphasizes the importance of modeling in acquiring new behaviors [29]. However, a heavy reliance on healthcare providers to model correct SI techniques may present a challenge in the context of workload and time constraints among clinic-based providers. Further, community-based providers are more likely to face a shortage of training materials, which hampers their ability to offer SI training to women [15]. Women seeking other contraceptive methods already feel that their local health systems are under-resourced and stretched too thin to provide quality counseling [41], implying that these constraints would be magnified in the case of SI which requires a lengthier counseling session than most of the other contraceptive methods. Thus, contraceptive SI programs need to be designed in a way that aligns women’s desire for comprehensive quality training with healthcare providers’ availability, keeping in mind that training content and delivery can influence who becomes a self-injector [10, 18].

### Environmental factors

Women’s strong desire to involve their intimate partners, family members, and friends in the contraceptive SI learning process underscores the importance of integrating social support into training programs to enhance confidence and facilitate a more inclusive environment for initiating and continuing with SI. This tallies well with one of the social cognitive theory’s principles that states external influencers from one’s environment can affect whether a learned behavior is maintained over time or not [29, 34, 42]. Research has consistently shown that strong social support positively influences women’s contraceptive decision-making [43–45]. Male partners, in particular, are motivated to support women to self-inject due to the perceived time and cost savings, convenience, and privacy [46]. This support can encompass helping with SI, providing reminders for re-injection dates, offering emotional encouragement, and accompanying women for counseling [46]. Some of our participants thought that SI referred to being injected by a lay (non-clinical) person. This is an area that needs further exploration to find out whether highlighting to women that they can enlist the help of others within their social networks to self-inject if they so wish, would increase overall interest in contraceptive SI.

Women's desire to bring their female relatives and friends into the SI learning journey, as highlighted in our study, reflects the substantial influence of these networks on family planning decisions. Women rely on their close social networks and peers to form their views of various contraceptive methods and ultimately choose a method [47, 48]. Healthcare providers and others looking to raise awareness or create a conducive environment for SI of DMPA-SC could leverage the power of social support to dispel myths and misconceptions and address the initial fear of contraceptive SI which may discourage women from opting for it despite its well-documented benefits.

The main strength of this study is that we enrolled women who had never used contraceptive SI thus gaining insight into their pre-conceived notions and the kind of support they felt they would need to self-inject successfully. These views are relevant to the designing of SI training for clients given that most women in Kenya will have had no prior SI experience and thus may hold similar learning preferences as expressed by our study participants. This paper presents a tight focus on a currently under-researched area, client SI learning, and complements the larger body of existing work on contraceptive SI. The study was not without limitations; for instance, we were not able to reach many married adolescents due to the legal restrictions on child marriages and hence these minors’ views may not be adequately represented. Second, the learning needs shared by participants were based on hypothetical use as these were not women seeking to self-inject and may differ from the views of women seeking SI services. Third, a detailed analysis exploring the differences in views between married and unmarried women was not carried out and therefore is not presented in this paper. Lastly, we only interviewed women in Kisumu and Nairobi and these may not be representative of the perspectives of most women in Kenya.

## Conclusion

Our study sheds light on non-SI users’ conceptualizations, preferences, and concerns regarding contraceptive SI training and their desire for a learning journey that delivers comprehensive information in a supportive environment. This has important implications for designing and implementing DMPA-SC SI training programs in Kenya. Rooted in the social cognitive theory, effective SI learning should integrate cognitive, behavioral, and environmental factors to impart knowledge, alleviate concerns about SI, and enhance self-efficacy. This can be achieved through observational learning from healthcare providers and providing ample SI practice opportunities for the client until she feels confident enough to continue self-injecting at home. By aligning with the WHO’s self-care guidelines and leveraging the social cognitive theory, we can develop a comprehensive approach that empowers women to confidently adopt SI should they so choose. Future initiatives should prioritize personalized, flexible SI learning approaches that cater to women's diverse needs and preferences, ensuring a supportive and enabling environment for contraceptive self-care.

## Supplementary Information

Supplementary Material 1.

Supplementary Material 2.

## Data Availability

The original data collection tools are included in the article as supplementary material. Further inquiries about the data can be directed to the corresponding author.

## Abbreviations

DMPA: Depot medroxyprogesterone acetate
ICAN: Innovations in Choice and Autonomy
KEMRI: Kenya Medical Research Institute
NACOSTI: National Commission for Science, Technology and Innovation
SC: Subcutaneous
SERU: Scientific and Ethics Review Unit
SI: Self-injection
UCSF: University of California, San Francisco
