# Supplementary material for: Knowledge, confidence and social support: Kenyan women’s priority needs for contraceptive self-injection learning through a social cognitive theory lens
Source: BMC Womens Health. 2025 Jun 30;25(Suppl 1):289. doi: 10.1186/s12905-025-03801-4 (PMC12207791; doi:10.1186/s12905-025-03801-4)
Supplement: Supplementary file 1 — Supplementary Material 1. [file 12905_2025_3801_MOESM1_ESM.docx]

**Appendix 1: Abbreviated In-Depth Interview Guide**

**Research Questions to be addressed through in-depth interviews:**

1. **Domain 1- Contraceptive decision-making**
   1. How do women form consciousness about contraceptive rights?
   2. How do women form contraceptive preferences?
   3. For whom might SI be a powerful method (even if they do not currently express interest)?
   4. How does women's consciousness of rights and preferences evolve over time? How do experiences acting on rights/preferences inform subsequent preferences and care-seeking? [Nigeria Cohort]
   5. How has the COVID-19 pandemic influenced women’s thoughts about and experiences with pregnancy and contraception?
2. **Domain 2- Contraceptive use:**
   1. What influences whether and how women can act on contraceptive rights and preferences?
   2. For whom might SI be a powerful method (even if they do not currently express interest)?
   3. How does women's consciousness of rights and preferences evolve over time? How do experiences acting on rights/preferences inform subsequent preferences and care-seeking? [Nigeria Cohort]
   4. How has the COVID-19 pandemic influenced women’s thoughts about and experiences with pregnancy and contraception?
3. **Domain 3- Self-injection**
   1. What are women's motivations and barriers for choosing SI, and what are the characteristics of women most interested in SI?
   2. Among SI users, what has their experience been with the method?
   3. What are women's preferences for SI provision/support?
   4. How do women's perspectives on SI change over time as the service provision landscape shifts? [Nigeria Cohort]
4. **Domain 4- Country-specific: E-commerce platform for Kenya**

**DOMAINS 1, 2 & 4 questions omitted here for simplicity. This paper reports findings from the highlighted questions**

Study Title: Innovations for Choice and Autonomy (ICAN)

IRB No: SERU 4013

Version: 4.1 DATED 03 FEB 21

Session ID: DMPA_[_____]_[___ ___ ]_[__]_[_____] Date (DD/MM/YY): ____ /____ / ____

Name of Interviewer: ___________________________________________________________

# Domain 3: Self-injection

[THIS SECTION IS MEANT TO UNDERSTAND WOMEN’S INTEREST IN SELF-INJECTION AND, FOR THOSE WHO HAVE EXPERIENCE, HOW IT HAS BEEN]

1. Are you aware of any medical/health products that you can inject by yourself at home (without the help of a medical provider or pharmacist)?
   1. **IF YES:**
      1. Which products are these?
      2. What do you know about these products?
      3. How did you hear about them?
      4. Are there any other products you are aware of?
      5. Have you ever used any of these products?
      6. Could you describe what this product looked like?

**PROCEED IF INTERVIEWEE HAS SELF-INJECTED DMPA-SC;
OTHERWISE SKIP TO QUESTION 53**

*Now I am going to ask you questions about Sayana Press. Sayana Press is an injectable family planning method that someone can buy from a pharmacy or get from a health facility and inject themselves every three months. It looks like this* ***[SHOW THE PARTICIPANT THE SAYANA PRESS MODEL]***

1. How many times have you injected yourself with Sayana Press?
2. When you received Sayana Press, what were you told about the possibility of injecting yourself?
   1. Were you told you could do the injection yourself?
   2. Were you offered the option of taking home doses to inject yourself with after your initial shot?
   3. What else were you told about the possibility of self-injection?
3. How has it been injecting yourself with Sayana Press?
4. What is easy about it?
5. What is difficult about it?
6. How has disposing of the needle been for you?
7. Were you taught how to inject yourself?
8. **IF YES**:
9. Who trained you?
10. Can you tell me how [trainer] taught you how to inject yourself?
11. What did the [trainer] say about how to know when to take your next dose?
12. What did the person say about disposing the needle?
13. What did you like about the training?
14. What didn’t you like about the training?
15. What additional information or support would have been helpful?
16. **IF NO**:
17. Why did you not receive training?
18. What additional information or support would have been helpful?

**IF PARTICIPANT IS USING SAYANA PRESS (DMPA-SC) BUT NOT SELF-INJECTING, GO TO QUESTION 43**

**INTERVIEWEE IS USING SAYANA PRESS AND IS SELF-INJECTING**

1. How does injecting yourself make you feel?
2. Why does it make you feel that way?
3. What does injecting yourself enable you to do that you couldn’t otherwise do if you had to go see a health provider each time?
4. Who in your life knows that you are self-injecting?
   - 1. Who else?
     2. Is there anyone you have kept it secret from?

**🡺 *[PROBE]: Why is that?***

- - 1. How have others in your life reacted to the fact that you are injecting yourself?
    2. How does this make you feel?

1. How confident do you feel that you could continue to inject yourself with Sayana Press if you wanted to?
   - 1. Why?
2. Do you plan to continue injecting yourself with Sayana Press?
   - 1. Why/why not?

**🡺 *[PROBE]:*** How does self-injection fit or not fit with your life right now?

- 1. **IF YES, PLANS TO CONTINUE SELF-INJECTING:**
     - 1. Where do you plan to get the refills next time?
       2. Why that person/place?
       3. What other help would you like in the future with injecting yourself?
       4. How many 3-month doses would you want to take home at a time?

**SKIP TO QUESTION 49**

**INTERVIEWEE IS USING SAYANA PRESS BUT NOT SELF-INJECTING**

1. How many times have you been injected with Sayana Press?
   1. Who has been injecting you?
   2. Is this every time you received a shot of Sayana?
   3. Why this person(s)?
   4. Would you consider being injected by another woman in your community that uses Sayana Press?
2. What has been your experience with being injected with Sayana Press?
   1. What has been easy?
   2. What has been hard?
3. Why have you not tried injecting yourself with Sayana Press?

a. Why do you feel that way?

b. What other concerns might you have about injecting yourself?

c. What help would you like to help you practice self-injection with Sayana Press?

i. Is there anything else?

1. Who in your life knows that you are using Sayana Press?
   1. Who else?
   2. Is there anyone you have kept it secret from?

**🡺 *[PROBE]:*** *Why is that?*

- 1. How have others in your life reacted to the fact that you are using Sayana Press, (*but not self-injecting*)?

**🡺 *[PROBE]:*** *Colleagues, friends, family, health worker*

- 1. How does this make you feel?

1. How confident do you feel that you will ever inject yourself with Sayana Press?
   1. Why or why not?
2. Do you plan to continue using Sayana Press?
   1. Why or why not?

**FOR ALL INTERVIEWEES USING SAYANA PRESS**

1. What are the benefits you see to self-injecting contraception rather than going to a health care provider or pharmacist for an injection?
   - 1. Any other benefits?
2. What are the challenges of self-injecting rather than going to a health care provider or pharmacist for an injection?
   - 1. Any other challenges?
3. Who do you think might most benefit from self-injectable contraception?
   - 1. Why this person/these people?
4. Would you recommend Sayana Press to other women?
   - 1. Why/why not?
     2. Do you think people you know would be interested in injecting themselves with Sayana Press?
        - 1. Why/why not?

**SKIP TO DOMAIN 4**

**IF THE INTERVIEWEE HAS NOT HEARD ABOUT SAYANA PRESS**

1. Scientists have developed a device that people can use at home to inject themselves with medicines like those that are used to treat diabetes **[SHOW UNIJECT DEVICE, DESCRIBE SELF-INJECTION].**  How would you feel about keeping medicines at home and injecting yourself with them when you needed them?
2. One use of these devices is contraception for pregnancy prevention. This is called “Sayana Press” and a person can inject themselves every three months to prevent pregnancy. If you needed contraception, would you be interested in injecting yourself with Sayana Press?
   - 1. Why/why not?

**🡺 *[PROBE]:*** Do you think people you know would be interested in injecting themselves with Sayana Press?

- - - - 1. Why/why not?
    1. **FOR CURRENT USERS OF ANY CONTRACEPTIVE METHOD:**
       - 1. Would you be interested in using Sayana Press instead of your current method?
         2. Why/why not?

**IF YES:**

- - - - 1. How much would you be willing to pay for Sayana Press?
        2. Where would you like to obtain Sayana Press?

1. If you wanted to inject yourself with Sayana Press, how would you like to learn how to inject yourself?

**🡺 *[PROBE]:*** *For example, going to the healthcare clinic, a pamphlet, online video/post*

1. Why would you like to learn this way?
2. Who would you like to be involved in helping you learn?
3. What are the questions you would want answered regarding self-injection?
4. How many 3-month doses would you want to take home at a time?
5. What are the benefits you see to self-injecting contraception rather than going to a health care provider or pharmacist for an injection?
   - 1. Any other benefits?
6. What are the challenges of self-injecting rather than going to a health care provider or pharmacist for an injection?
   - 1. Any other challenges?
7. Who do you think might most benefit from self-injectable contraception?
   - 1. Why this person/these people?
