# Supplementary material for: Knowledge, confidence and social support: Kenyan women’s priority needs for contraceptive self-injection learning through a social cognitive theory lens
Source: BMC Womens Health. 2025 Jun 30;25(Suppl 1):289. doi: 10.1186/s12905-025-03801-4 (PMC12207791; doi:10.1186/s12905-025-03801-4)
Supplement: Supplementary file 2 — Supplementary Material 2. [file 12905_2025_3801_MOESM2_ESM.docx]

**Appendix 2 Description of self-injection of DMPA-SC provided to IDI participants**

Participants were shown a sample DMPA-SC Unit and also viewed a publicly available video developed by John Snow Inc. international to demonstrate how to self-inject DMPA-SC. The video is entitled “How to give yourself an injection with Subcutaneous DMPA (DMPA-SC): An all-in-one contraceptive” and is available at this link (<https://www.youtube.com/watch?v=9iyVsFNZnuw>).
